# A 70-Year-Old Man with a Rare Type of Gastric Cancer

**DOI:** 10.34172/mejdd.2024.380

**Published:** 2024-04-30

**Authors:** Iman Soufi Afshar, Naghmeh Salarieh, Pardis Ketabi Moghadam, Arash Daryakar

**Affiliations:** ^1^Research Institute for Gastroenterology and Liver Diseases, Shahid Beheshti University of Medical Sciences, Tehran, Iran

 According to the estimate of GLOBOCAN, more than 800 000 deaths are attributed to gastric cancer and more than 1 100 000 new cases of gastric cancer were detected worldwide in 2020. About 75% of all deaths and new cases are reported in Asia.^1^ The two principal subtypes of gastric adenocarcinoma, including intestinal type and diffuse type, have been elaborately explained. Intestinal type adenocarcinoma is stratified into three subgroups: papillary, tubular, and mucinous. Diffuse-type adenocarcinoma comprises signet-ring cell carcinoma and other poorly cohesive carcinomas.^2^ A small part of gastric cancers is classified as indeterminate type. They consist.^2^ Gastric adenosquamous carcinoma is a rare type of gastric cancer, which is mostly discovered in Asian men in the sixth decade of.^3^ Diagnosis is confirmed when the histological features of adenocarcinoma and squamous cell carcinoma are found in the specimen.^4^ The squamous component should contain more than 25% of the primary tumor.^5^ The absence of adenosquamous cell carcinoma in any other organ is another requirement for the diagnosis of gastric adenosquamous cancer. Whether gastric adenosquamous cell carcinoma is originally adenosquamous or is a metaplastic transformation of adenocarcinoma into ectopic squamous cell carcinoma is still under debate. Merging of concomitant adenocarcinoma and squamous cell carcinoma ^6^ and dual stem cell differentiation toward both glandular and squamous carcinomas are other proposed hypotheses.^7^ TNM staging (evaluating tumor depth, lymph node involvement, and distent metastasis) appears to be indicative of overall survival.^3^ The survival rate of gastric adenosquamous carcinoma is not favourable since it usually presents at advanced stages while invading the muscularis propria or beyond. The liver is the common site of metastasis.^3,4^ Metastatic lymph node contribution from adenocarcinoma or squamous cell carcinoma may impact the prognosis since the presence of adenocarcinoma predicts a better outcome. Most studies declare that the survival rate hardly exceeds 24 months.^3^ A 70-year-old man with a positive history of iron deficiency anemia from 8 months earlier was referred to the gastroenterology clinic of Lahijan, Guilan, Iran, for further evaluation. His medical history and family history were unremarkable except for hypertension. Just like other Gilaki people residing in the northern part of Iran, his nutritional habits were noticeable for overconsumption of pickles, salty and smoked food, as well as high temperature drinks. Total colonoscopy was normal. On endoscopy, a large round infiltrative, ulcerative, and necrotic mass-like lesion was detected in incisura angularis (Figure 1). Multiple biopsies were taken, which were suggestive of poorly differentiated adenosquamous carcinoma. Neither lymphovascular invasion nor perineural space involvement was identified in the specimens (Figure 2). As can be seen in Figures 3 and 4, the immunohistochemistry (IHC) study of the received specimens was positive for expression of tumor protein p63 encoded by Tp63 gene and CK5-6 expressed by epithelial cells in the squamous portion of the tumor. CK7 expressed by epithelial cells and CDX2 expressed in the nuclei of intestinal epithelial cells were indicative of the adenocarcinoma component of the tumor. PAS-positive mucin was also observed in the adenocarcinoma section of the tumor. As driven from Figure 5, the specimen presented a high ki67 labelling code. An abdominopelvic computed tomography (CT) scan was performed, which revealed an asymmetric wall thickening of the gastric body along with a few regional lymph nodes with a maximum short-axis diameter of 12 mm (Figure 6). No distant metastasis, peritoneal involvement, and ascites were detected in further evaluations. Given the imaging, it was estimated to have at least a T3N1M0 primary gastric cancer. The patient proceeded with total gastrectomy, lymph node dissection, and adjuvant chemotherapy. The surgical specimen revealed a 65 × 50 × 23 mm ulcerative lesion extending circumferentially from greater curvature of the body into the anterior and posterior walls of the body with nodular involvement of subserosa, safe macroscopic and microscopic margins from omentum and visceral peritoneum, 7 involved lymph nodes out of 28 resected lymph nodes, and free surgical margins consistent with T3N3aM0. Adjuvant chemotherapy was completed with FOLFOX regimen including folinic acid, 5-fluorouracil, and oxaliplatin, which seemed to be active against both adenocarcinoma and squamous cell carcinoma components of the tumor. Radical resection is still the preferred treatment for local disease without distant metastasis. Although no standard adjuvant therapy strategies for gastric adenosquamous carcinoma have been established, chemotherapy has been considered to be useful for gastric adenosquamous carcinoma.^8^ Radiotherapy could be introduced as another method of treatment since the squamous component of gastric adenosquamous carcinoma is sensitive to radiotherapy.^3^ The 6-month follow-up of the presented patient after radical resection and FOLFOX regimen did not reveal any evidence of disease recurrence, confirming treatment efficacy in patients without distant metastasis.

**Figure 1:**
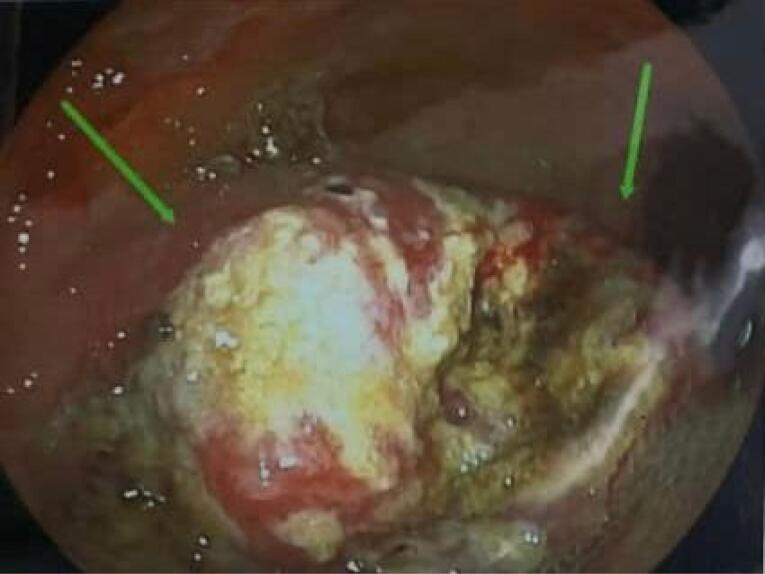
Endoscopic view of gastric adenosquamous carcinoma in the presented case revealed a large necrotic and ulcerative mass-like lesion covered by a white mucous membrane (The tumor is pointed by arrows)

**Figure 2:**
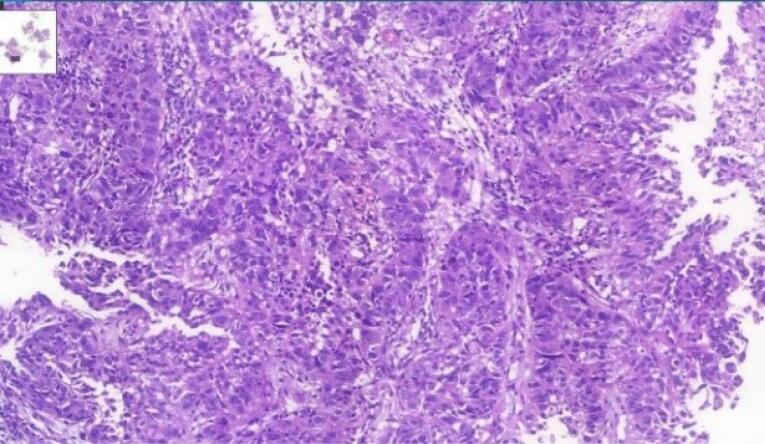
Hematoxylin and Eosin staining reveals an admixture of arrangements of glandular adenocarcinoma component and squamous cell carcinoma component consistent with poorly differentiated gastric adenosquamous carcinoma in the received specimens. Keratin formation is evident in the squamous cell carcinoma (SCC) component

**Figure 3:**
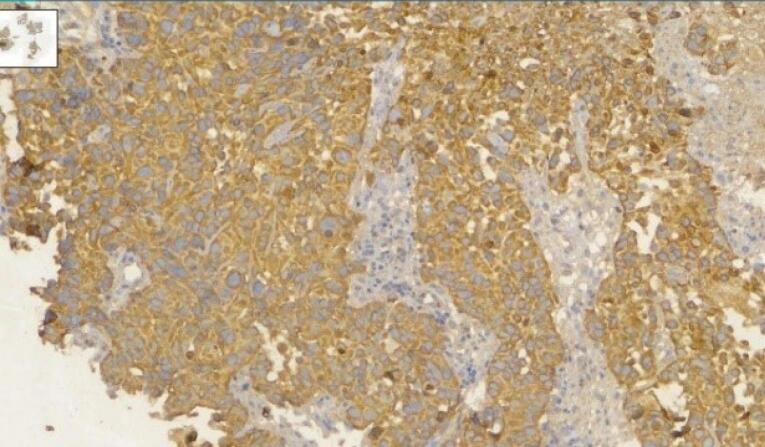
Immunohistochemistry (IHC) study of the received specimens was positive for CK 5,6 expressed by epithelial cells in the squamous portion of the tumor, CK7 expressed by epithelial cells, and CDX2 expressed in the nuclei of intestinal epithelial cells indicative of adenocarcinoma component of the tumor (Brown color shows strong expression of mentioned CK)

**Figure 4:**
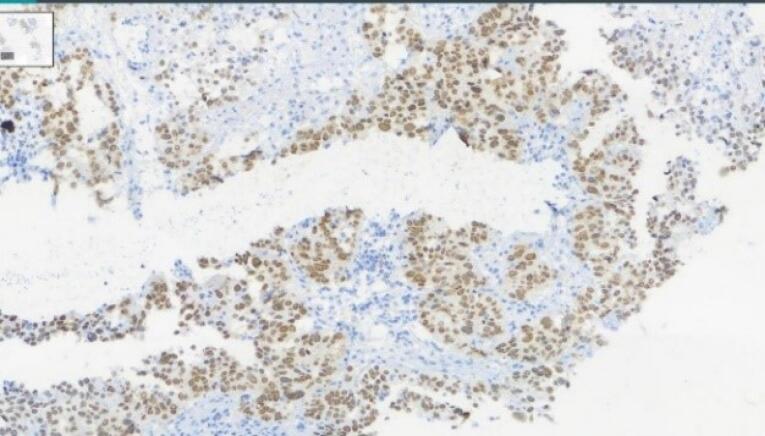
IHC study of the received specimens was positive for expression of tumor protein p63 encoded by Tp63 gene in the squamous portion of the tumor (Brown indicates positive cells)

**Figure 5:**
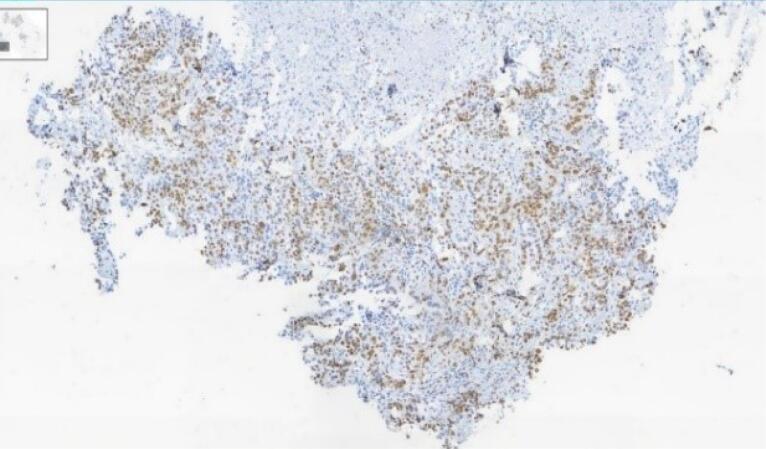
The high labelling code of Ki67 is evident in the received specimen (Brown indicates positive cells)

**Figure 6:**
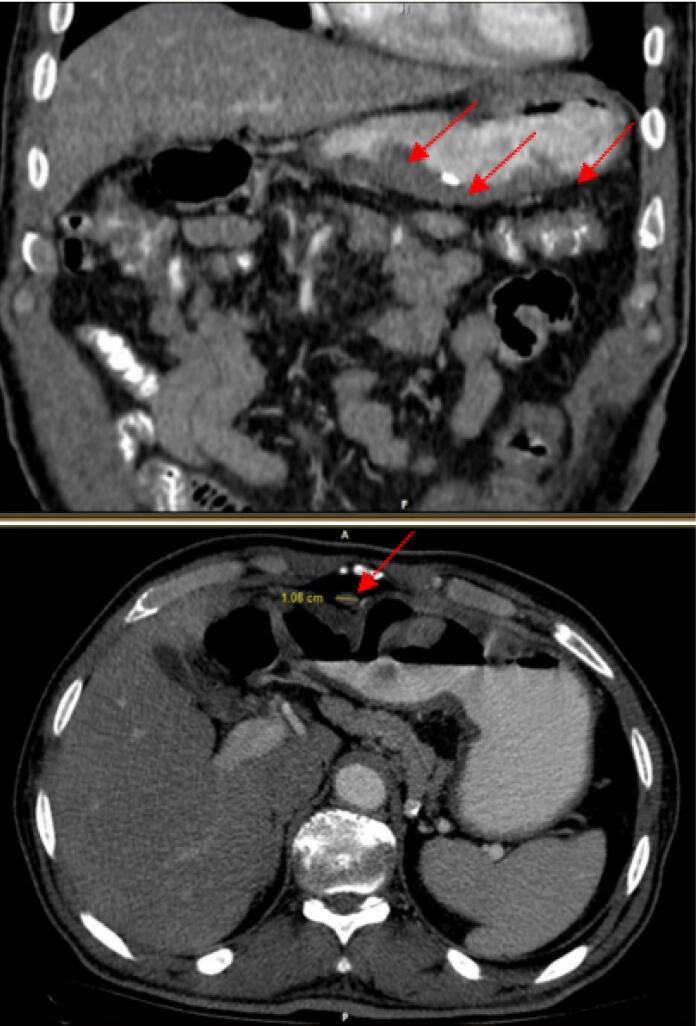
Asymmetric gastric wall thickening particularly in greater curvature of body is seen in the coronal view of the abdominopelvic tomogram. A 1.1 cm regional lymph node has been pointed in the axial plane of abdominopelvic tomogarm. The abnormalities were marked by arrows to make them more prominent.

## What is your diagnosis?

 Answer: Diagnosis: Gastric adenosquamous carcinoma
